# Potent inhibition of tumoral hypoxia-inducible factor 1α by albendazole

**DOI:** 10.1186/1471-2407-10-143

**Published:** 2010-04-15

**Authors:** Mohammad H Pourgholami, Zhao Y Cai, Samina Badar, Kiran Wangoo, Marianne S Poruchynsky, David L Morris

**Affiliations:** 1 Cancer Research laboratories, University of New South Wales Department of Surgery, St George Hospital, Sydney, NSW 2217, Australia; 2 Medical Oncology Branch, National Cancer Institute, Bethesda, Maryland, USA

## Abstract

**Background:**

Emerging reports suggest resistance, increased tumor invasiveness and metastasis arising from treatment with drugs targeting vascular endothelial growth factor (VEGF). It is believed that increased tumoral hypoxia plays a prominent role in the development of these phenomena. Inhibition of tumoral hypoxia inducible factor (HIF-1α) is thus becoming an increasingly attractive therapeutic target in the treatment of cancer. We hypothesized that the anti-VEGF effect of albendazole (ABZ) could be mediated through inhibition of tumoral HIF-1α.

**Method:**

*In vitro*, the effects of ABZ on HIF-1α levels in human ovarian cancer cells (OVCAR-3) were investigated using hypoxic chamber or desferrioxamine (DFO) induced-hypoxia. *In vivo*, the effects of ABZ (150 mg/kg, i.p., single dose) on the tumor levels of HIF-1α and VEGF protein and mRNA were investigated by western blotting, RT-PCR and real time-PCR.

**Results:**

*In vitro*, ABZ inhibited cellular HIF-1α protein accumulation resulting from placement of cells under hypoxic chamber or exposure to DFO. *In vivo*, tumors excised from vehicle treated mice showed high levels of both HIF-1α and VEGF. Whereas, tumoral HIF-1α and VEGF protein levels were highly suppressed in ABZ treated mice. Tumoral VEGFmRNA (but not HIF-1αmRNA) was also found to be highly suppressed by ABZ.

**Conclusion:**

These results demonstrate for the first time the effects of an acute dose of ABZ in profoundly suppressing both HIF-1α and VEGF within the tumor. This dual inhibition may provide additional value in inhibiting angiogenesis and be at least partially effective in inhibiting tumoral HIF-1α surge, tumor invasiveness and metastasis.

## Background

Amongst the vast array of proangiogenic molecules identified, VEGF has been shown to play a pivotal role in tumor angiogenesis. VEGF is a potent stimulator of endothelial cell survival, mitogenesis, migration and differentiation [1,2]. Angiogenesis inhibitors targeting VEGF have shown anticancer activity in preclinical and clinical trials. Several VEGF inhibitors have been approved by the US Food and Drug administration for the treatment of tumors or age-related macular degeneration [3,4]. However, recently emerging reports suggest that the effects of these drugs in cancer are only transitory, not producing enduring efficacy in terms of either tumor shrinkage or dormancy or long term survival, thus resulting in eventual drug resistance, vascular recovery and relapse to progressive tumor growth [5-7]. A number of contributing mechanisms have been proposed including up-regulation of fibroblast growth factor (FGF), matrix metalloproteinases (MMPs) and the induction of HIF-1α [6,8]. The most important mediator identified to date of the cell's response to reduced oxygen availability, HIF-1α is preserved and activated in response to reduced oxygen availability [9]. By affecting the expression of a wide array of genes, HIF-1α plays a central role in angiogenesis and in regulating the adaptation and survival of tumors [10,11]. Perhaps to some extent this is because transcriptional regulation of VEGF is critically dependent on HIF-1α. Results from several studies have provided compelling evidence that hypoxia-triggered up-regulation of other proangiogenic factors in the presence of anti-VEGF agents can restimulate tumor angiogenesis through VEGF-dependent or VEGF independent pathways [6,12,13]. More importantly, HIF-1α induction by hypoxia has been associated with the emergence of a more aggressive tumor phenotype [14]. On this basis, drugs that inhibit VEGF and angiogenesis through the inhibition of HIF-1α may provide therapeutic benefit over those which target the VEGF or its signaling pathway only.

Albendazole, methyl 5-propylthio-1H-benzimidazole-2-yl carbamate, is a benzimidazole carbamate originally developed as a veterinary product back in 1975 and despite its extensive use in man and farm animals, few adverse events have been associated with its use [15,16]. Its anthelmintic action has been attributed to binding to the helminth β-tubulin, leading to depolymerization, cell cycle arrest and death [17,18]. Because of their interaction with the microtubules, in recent years, benzimidazole carbamates such as albendazole and mebendazole have been under investigation as anticancer agents. Cell culture and animal studies utilizing human cancer cells have revealed that both these agents are potent inducers of apoptosis and inhibitors of tumor growth [19-23]. More recently, using an experimental model of ovarian cancer with malignant ascites formation, we demonstrated that, chronic treatment with ABZ leads to suppression of VEGF levels, inhibition of malignant ascites formation and arrest of tumor growth [24,25]. In the current study we sought to investigate if the observed ABZ anti-VEGF effect is mediated through the inhibition of the HIF pathway. Herein, we report that, *in vitro *experiments performed using hypoxic chamber and DFO, provided evidence for the anti-HIF-1α activity of ABZ. Then, study of the effects of a single dose of ABZ on tumoral HIF-1α and VEGF expression revealed profound suppression of both HIF-1α and VEGF protein levels. Thus, results from this study demonstrate that ABZ is a potent inhibitor of HIF-1α under both *in vitro *and *in vivo *conditions.

## Methods

### Chemicals and antibodies

Unless otherwise stated, all drugs and chemicals used in this study were obtained from Sigma-Aldrich (Australian subsidiary, Sydney). The following primary antibodies were used through out this study: HIF-1α (H-206) rabbit polyclonal IgG (Santa Cruz Biotechnology), VEGF (C-1) mouse monoclonal IgG (Santa Cruz Biotechnology, Sydney, Australia.), Monoclonal anti-β-actin (Sigma-Aldrich). Secondary antibodies were goat anti rabbit IgG HRP (Santa Cruz Biotechnology) and anti mouse IgG peroxidase (Sigma-Aldrich).

### Cell culture

The human ovarian cancer cells (OVCAR-3), originally obtained from the American Type Culture Collection (ATCC) were prepared for *in vitro and in vivo *growth experiments as previously described [24]. Cells were maintained in RPMI 1640 medium with 2 mM l-glutamine, 2 g/L sodium bicarbonate, 4.5 g/L glucose, 10 mM HEPES, 1 mM sodium pyruvate, 0.01 mg/mL bovine insulin, supplemented with 100 units/mL penicillin and 100 units/mL streptomycin and 10% FBS in a humidified atmosphere at 37°C.

### In vitro hypoxia tests

OVCAR-3 cells (3 × 10^6^) seeded in 75 cm^2 ^flasks were grown for 72 h at 37°C to 80% confluency. Cells were treated with ABZ (0 -1 μM) before being placed in a sealed modular hypoxic chamber (Billups-Rothenburg, Del Mar, CA) flushed with 1% O2, 5% CO2 and 94% N2. The chamber was then placed in an incubator at 37°C for 4 h. For induction of chemical hypoxia, the same procedure was used, except that instead of placement in hypoxic chamber, cells were treated with the chemical hypoxic agent desferrioxamine (DFO, 100 μM) for 4 h. DFO is a well established hypoxymimetic agent [26]. Cells not exposed to hypoxia or DFO were run in parallel as controls. Following treatment, cells were washed with PBS and scraped into RIPA buffer (300 μL). Lysates were centrifuged (8000 rpm, 4°C, 10 min) and stored at -80°C for analysis. HIF-1α and VEGF protein expressions were determined by western blot analysis, while RT-PCR and real time PCR were used to determine mRNA levels.

### Establishment of i.p. xenograft

Intraperitoneal tumors were grown in 6 week old female nude athymic Balb C nu/nu mice (Animal Resources Centre, Perth, Western Australia). Each mouse was injected i.p. with 10 million OVCAR-3 cells suspended in 1 mL of the medium. Animals were housed under complete aseptic conditions, fed autoclaved pellets and sterile water ad libitum. Health status of each animal was monitored daily and all animal procedures were conducted in conformity with institutional animal ethics committee guidelines (University of New South Wales, Sydney, Australia).

### Drug treatment

Three weeks post cell inoculation, animals were randomly assigned to one of the 6 treatment groups (6 mice/group). Before proceeding with drug treatment, animals were subjected to peritoneal lavage (2 mL of sterile normal saline injected i.p. and aspirated immediately after kneading). Mice were then immediately treated i.p. (1 mL/20 g body weight) with either the vehicle [0.5% w/v hydroperoxymethyl cellulose (HPMC)] or ABZ (150 mg/kg suspended in HPMC) followed by euthanasia at the predetermined time. Group 1 animals were euthanized immediately after vehicle treatment. Groups 2-6 were treated with ABZ and euthanized at 1, 6, 24, 48 or 72 h post injection respectively. Following euthanasia, peritoneal cavity was washed, tumors were excised, rapidly snap frozen in liquid nitrogen and stored at -80°C for subsequent analysis. Tumors were analysed for the expression of VEGF and HIF-1 protein and mRNA.

### Western blot analysis

Equivalent amounts of whole cell extracts (or tumor protein) were resolved in SDS PAGE (10% for HIF-1α and 12% for VEGF) and transferred to a PVDF membrane. The membrane was blocked in 5% non-fat dry milk in TBST and incubated (2 h) with indicated primary antibodies (HIF-1α and VEGF, 1:200), followed by incubation (1 h) with secondary anti body, goat anti-rabbit (1:5000) or anti-mouse secondary antibody (1:160,000) (Sigma-Aldrich;) for HIF-1α and VEGF respectively. Immunoreactivity was visualised by enhanced chemiluminescence reagent (Perkin Elmer Cetus, Foster City, CA, USA). To demonstrate equal loading, blots were stripped and reprobed with a specific antibody recognizing β-actin (1:5000 dilution; Sigma-Aldrich).

### Determination of mRNA by RT-PCR

Mice tumor extracts were examined for the expression of VEGF and HIF-1α mRNA by reverse transcription-PCR. Total RNA was isolated from the cells using the highly pure RNA isolation kit according to the protocol provided by the manufacturer (Invitrogen, Sydney, Australia). Primers for the amplification of VEGF and HIF-1α were constructed based on the following sequence: VEGF sense: 5,-CAC ATA GGA GAG ATG AGC TTC-3; VEGF anti-sense: 5,-CCG CCT CGG CTT GTC ACA T-3. The primer amplifies the various VEGF isoforms (121 to 206). HIF-1α sense primer 5,-TCA AAG TCG GAC AGC CTC A-3, HIF-1α anti-sense: 5,-CCC TGC AGT AGG TTT CTG CT-3 product for 460 bp,. The β-actin gene was used as an internal control (202 bp; β-actin sense: 5'-CTT CCT GGG CAT GGA GTC CT-3'; β-Actin anti-sense: 5'-GGA GCA ATG ATC TTG ATC TT-3'). Total RNA was used to amplify HIF-1α/VEGF/β-actin using the Superscript TM One Step RT-PCR with Platinum^®^Taq (Invitrogen, Sydney, Australia). The amplification was carried out using a Palm Cycler after an initial cDNA synthesis at 54°C for 30 min and 5 min at 94°C for denaturation. This was followed by 27 cycles of denaturation at 94°C for 1 min, primer annealing at 60°C for 1 min, primer extension at 72°C for 45 s and a final extension of 72°C for 10 min. The RT-PCR products were visualized by electrophoresis (45 min at 100 V) on 1.5% agarose gel in 1 × TAE buffer containing ethidium bromide.

### Determination of mRNA by Real-Time PCR

To quantify mRNA levels, real-time RT-PCR (qRT-PCR) was used. Briefly, RNA was isolated as described above using TRIzol reagent. cDNA synthesis was performed on RNA (1 μg) using Super Script III First - Strand Synthesis Super Mix kit (Invitrogen Life Technologies). Real time was performed in a Roter Gene 3000 (Corbett Life Science, Mortlake, Australia) using SYBR Green ER qPCR Super Mix Universal kit (Invitrogen life technologies) as per supplier protocol. Primers used to amplify specific gene products were GAPDH Sense 5'GCG CTG AGT ACG TCG TGG AG 3' GAPDH Antisense 5'CAG TTG GTG GTG CAG GAG GAG G-3'; HIF-1α Sense 5'-CCA GTT ACG TTC CTT CGA TCA GT-3'; Anti sense 5'-TTT GAG GAC TTG CGC TTT CA-3'. Amplification reaction used for HIF-1 were performed as previously described [27]. Data obtained are expressed as CT which is the PCR cycle number at which the accumulated fluorescent signal in each reaction crosses a threshold above background. The relative expression levels were calculated relative to the control using the comparative Ct (ΔΔCt) method where the relative expression is calculated as 2-ΔΔCt.

### Statistical analysis

GraphPad Prism version 5.0 was used for data analysis. All data are reported as the mean ± s.e.m. *In vitro *data were analysed using Student's *t *test followed by Tukeys. Animal data were analysed using Mann-Whitney U test. Effects were considered to be statistically significant at p < 0.05.

## Results

### ABZ does not affect HIF-1α under normoxic conditions

**Figure 1:**
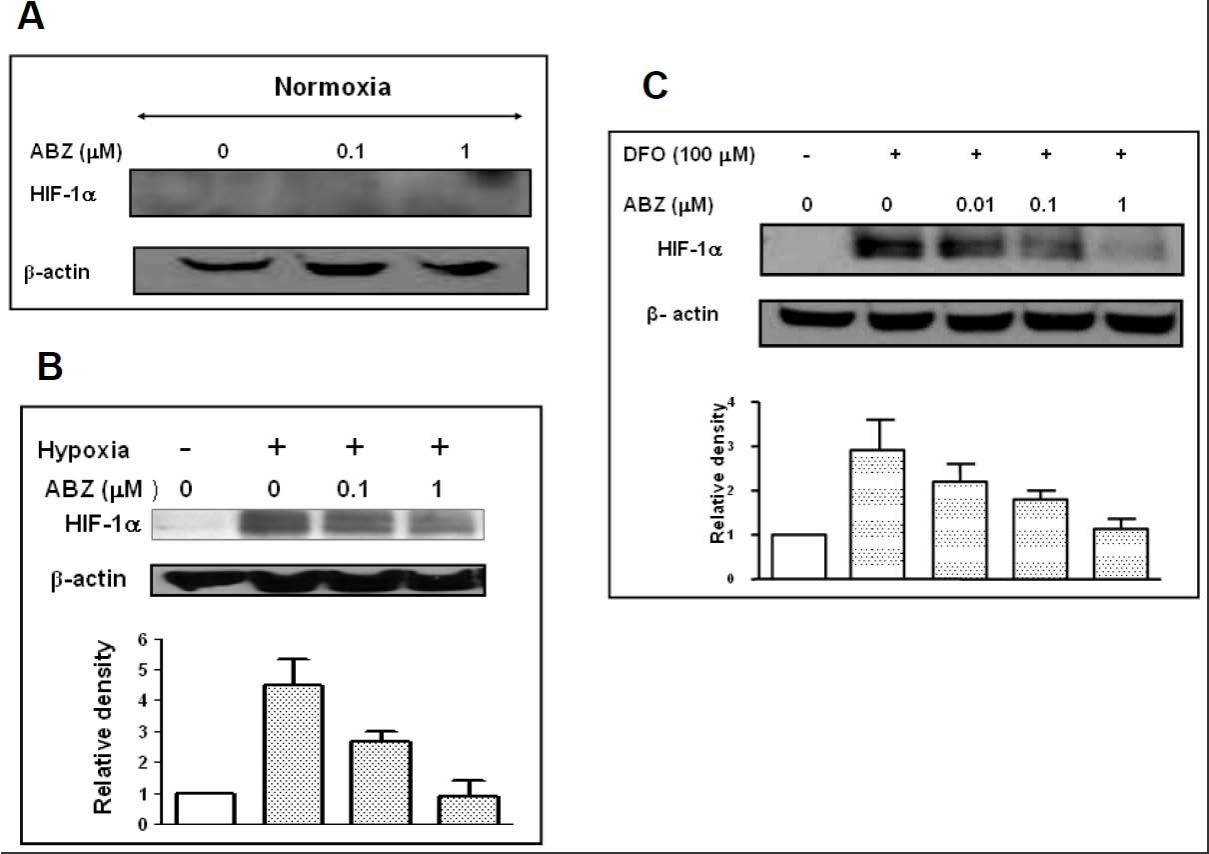
**Effect of ABZ on HIF-1α expression in vitro**. Albendazole inhibits *in vitro *HIF-1α protein expression induced by hypoxia. OVCAR-3 cells incubated with increasing concentrations of ABZ were either kept under normoxic conditions (A) or were transferred to hypoxic chambers for 4 h (B). At the end of the experiment, whole cell extracts were examined for the expression of HIF-1α protein. Similarly, cells treated with ABZ were incubated under cell culture conditions with 100 μM hypoxia-inducing agent desferrioxamine (DFO) for 4 h (C). Each experiment was repeated at least twice. Values (mean ± s.e.m) were normalized to β-actin and the control vehicle treated cells.

We first examined the effect of ABZ on HIF-1α under normoxic cell culture conditions. Here, OVCAR-3 cells grown in culture were treated with ABZ (0.1-1 μM) for 4 h and then examined for the expression of HIF-1α protein. The cells did not express measurable quantity of HIF-1α protein under normoxic conditions and their treatment with ABZ had no affect on HIF-1α protein expression (Figure 1A).

### ABZ treatment inhibits hypoxia-induced accumulation of HIF-1α protein in vitro

We next examined the effect of ABZ on hypoxia-induced HIF-1α accumulation *in vitro*. ABZ treated cells placed in hypoxic chamber for 4 h were analysed for the expression of HIF-1α. Hypoxic conditions led to dramatic increase in HIF-1α levels while, pre-treatment of cells with ABZ inhibited HIF-1α accumulation in a dose-dependent manner (Figure 1B). Under these conditions, and compared to vehicle treated cells, the HIF-1α levels were reduced by 41% (p < 0.05) and 79% (p < 0.001) in 0.1 and 1 μM ABZ treated cells respectively.

Utilizing DFO, chemically induced hypoxia was used to obtain further evidence on the *in vitro *effect of ABZ on cellular HIF-1α expression. Exposure of cells to the hypoxia mimetic agent DFO, led to 3 fold increase in HIF-1α protein expression. Pre-treatment of cells with ABZ led to concentration-dependent reduction in HIF-1α levels (Figure 1C). Compared to vehicle treated controls, HIF-1α protein content in cells exposed to the 1 μM ABZ were profoundly reduced (p < 0.001).

### ABZ treatment diminishes tumoral HIF-1α expression

**Figure 2:**
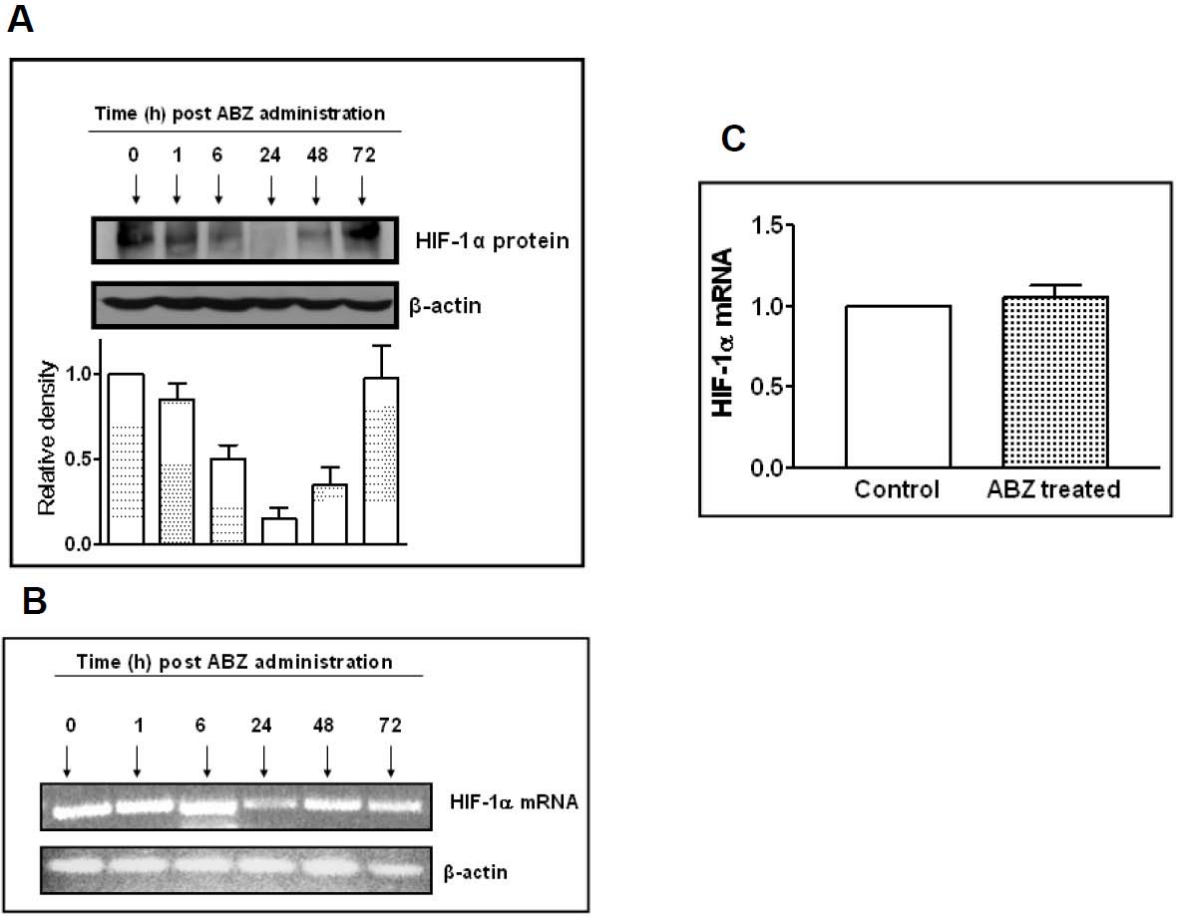
**Suppression of tumoral HIF-1α by ABZ**. Tumors harvested at various time-points from ABZ treated mice (single dose 150 mg/kg; i.p.) were examined by western blot analysis for the expression of HIF-1α protein (A) or by means of RT-PCR (B) and real time- PCR (C) for HIF-1α mRNA expression. Figures are representatives of protein and mRNA expression from the same tumor (6 mice per each treatment group).

In order to determine if these *in vitro *observations do translate into *in vivo *anti-HIF effects, tumor bearing mice were given a single dose of ABZ and their tumors were excised at various time-points post drug administration. Using western blot analysis, HIF-1α levels in these tumors were then assessed. As shown in Figure 2A, tumoral HIF-1α protein levels were highly reduced for up to 48 h post ABZ administration. Peak ABZ anti-HIF-1α effect was found to be in tumors harvested at 24 h post drug administration. To find out if the HIF-1α suppression was a biological consequence of HIF-1mRNA inhibition by ABZ, using RT-PCR, the tumor samples were examined for HIF-1mRNA expression. Except for the 24 h treated tumors, no reduction in HIF-1mRNA could be detected (Figure 2B). To verify this, the 24 h tumor samples were further analysed by real-time PCR. Results obtained show no difference in tumoral HIF-1mRNA expression between the vehicle and ABZ treated groups (Figure 2C).

### ABZ inhibits tumoral accumulation of VEGF protein and mRNA

**Figure 3:**
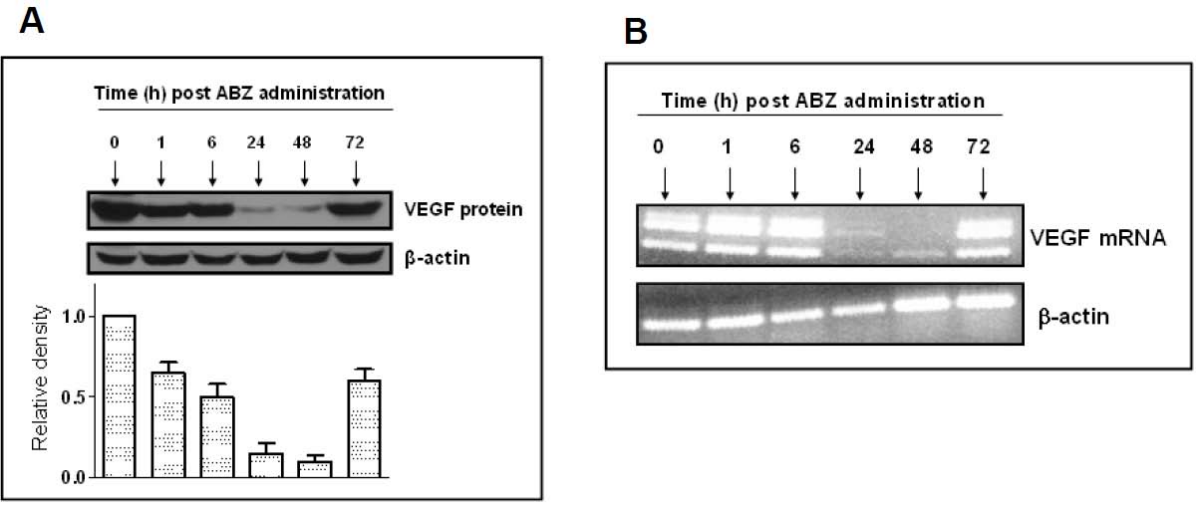
**ABZ inhibits tumoral VEGF protein and mRNA**. In mice bearing intra peritoneal OVCAR-3 tumors, acute treatment with albendazole (150 mg/kg, i.p.) led to time-dependent suppression of tumoral VEGF levels. The peak anti-VEGF effect was observed in tumors harvested 24 to 48 h post drug administration (A). RT-PCR analysis of the tumor samples revealed complete depletion of VEGFmRNA in these tumors (B).

VEGF expression is tightly controlled by HIF-1α, thus a reduction in HIF-1α expression should be directly reflected in VEGF expression. We therefore examined the tumor tissues for the expression of VEGF. As depicted in Figure 3A, VEGF is highly expressed in these tumors and treatment with a single dose of ABZ led to dramatic and time-dependant decline on tumoral VEGF levels. This effect paralleled the time-dependent suppression of HIF-1α with the ABZ effect peaking during the 24-48 h post drug administration period (in 24 and 48 h samples p < 0.001 compared to zero time controls). To confirm that the ABZ effect on the VEGF levels resulted from inhibition of transcriptional activity, tumoral VEGFmRNA levels were examined by RT-PCR. As illustrated in Figure 3B, VEGFmRNA levels were highly suppressed in the 24 and 48 h tumor samples. These results provide strong evidence in support of anti-HIF activity of ABZ *in vivo *and hence reveal the mechanism behind its potent anti-VEGF effects.

## Discussion

Chronic ABZ treatment has recently been shown to produce antitumor and anti-VEGF effects. The results from the present study reveal that ABZ inhibits HIF-1α accumulation and VEGF production after acute administration. We examined the effects of ABZ on HIF-1α levels in cell culture under both normoxic and hypoxic conditions. Although the HIF-1α gene is constitutively transcribed, under normoxic conditions HIF-1α protein is virtually undetectable due to its oxygen-dependent degradation by the propyl hydroxylases [28]. However, exposure to hypoxia stops HIF-1α degradation and hence leads to its rapid accumulation in the cell [29]. Similarly, treatment of cells with DFO (an inhibitor of propyl hydroxylases) also causes cellular accumulation of the HIF-1α protein [26]. Herein, we found that pre-treatment of OVCAR-3 tumor cells with ABZ inhibits hypoxia or DFO-induced HIF-1α accumulation. More importantly, the present study revealed that, *in vivo *administration of ABZ as a single dose leads to profound suppression of tumoral HIF-1α and VEGF.

It has been well established that solid tumor growth is angiogenesis dependent and hypoxia is the major pathophysiologic condition that regulates angiogenesis [10]. Tumor hypoxia arises as a result of increased metabolic activity and oxygen consumption by the rapidly proliferating tumor cells. HIF-1α is a transcriptional activator that mediates adaptive responses to hypoxia through the induction of a number of growth factors and cytokines [2,9]. In solid tumors, HIF-1α is a potent inducer of VEGF which then plays a pivotal role in the process of angiogenesis [30-32]. Based on this, targeting tumoral HIF-1α is under intense investigation as a therapeutic strategy to inhibit angiogenesis and tumor growth.

In rapidly growing solid tumors reduced oxygen availability leads to inhibition of propyl hydroxylases, a dramatic surge in HIF-1α protein levels and consequently induction of VEGF mRNA and protein [33]. Up-regulation of VEGF leads to a series of events culminating in the formation of new vessels to support the rapidly dividing cells with the necessary oxygen and nutrients [9,34]. In addition to up-regulation of VEGF expression, HIF-1α itself has been found to be an important mediator of survival and angiogenesis and its over expression in the majority of the human cancers has been associated with patient mortality and poor response to treatment [35,36]. Based on this, targeting tumoral HIF-1α is under intense investigation as a therapeutic target for cancer chemotherapy.

Furthermore, emerging experimental and clinical data from VEGF-targeted therapies are suggesting development of resistance and increased tumor invasiveness and metastasis [6,12,37]. While it is entirely possible that the resistance mechanisms are diverse and depend on tumor type and the drug employed, up-regulation of several well-defined signaling pathways such as placental growth factor (PIGF), fibroblast growth factor (FGF), matrix metalloproteinases (MMPs), notch and the HIF pathway have been suggested [6,8]. In line with this, recent studies have implicated the hypoxia/HIF-1α as an instigator of invasion and metastasis [14,38,39]. Thus tumoral HIF-1α inhibition is becoming an increasingly attractive therapeutic target in the treatment of cancer [40].

In this study we demonstrate for the first time that ABZ is a potent inhibitor of HIF-1α under both *in vitro *and *in vivo *conditions. Under cell culture conditions, ABZ inhibited hypoxia and DFO-induced accumulation of HIF-1α. *In vivo*, ABZ treatment led to diminished tumoral HIF-1α levels. In line with this, tumoral VEGF levels were also profoundly suppressed. Further trials are needed to show if inhibition of the HIF-VEGF axis by ABZ provides additional therapeutic benefit over agents that only inhibit VEGF or it's down stream signalling pathways.

## Conclusion

ABZ, a benzimidazole carbamate with extensive clinical use as an anthelmintic, was shown in this study to be a potent inhibitor of tumoral HIF-1α. Both VEGF and HIF-1α are thought to be crucial mediators of angiogenesis and tumor growth. Anti-VEGF agents may induce tumor resistance. Inhibition of tumoral HIF-1α by ABZ may thus prove to be advantageous in HIF induced anti-VEGF resistance.

## Competing interests

The authors declare that they have no competing interests.

## Authors' contributions

The author MHP designed the investigation, analysed the data and prepared the manuscript. The authors ZYC, SB and KW performed the experiments and also took part in data analysis and discussions. Authors, MSP and DLM provided important input into designing experiments, review and editing of the manuscript. All authors read and approved the final version of the manuscript.

## Pre-publication history

The pre-publication history for this paper can be accessed here:

http://www.biomedcentral.com/1471-2407/10/143/prepub
